# The Chinese medicine Chai Hu Li Zhong Tang protects against non-alcoholic fatty liver disease by activating AMPKα

**DOI:** 10.1042/BSR20180644

**Published:** 2018-11-07

**Authors:** Meng Zhang, Yuan Yuan, Qing Wang, Xiaobo Li, Jiuzhang Men, Mingxin Lin

**Affiliations:** 1 Basic Medical College, Shanxi University of Chinese Medicine, Jinzhong, China; 2 Department of Chinese Medicine, First Hospital of Shanxi Medical University, Taiyuan, China; 3 Institute of Basic Theory of Traditional Chinese Medicine, China Academy of Chinese Medicine Science, Beijing, China

**Keywords:** AMPKα, Chinese medicine, non-alcoholic fatty liver disease, PPAR-γ

## Abstract

An effective treatment for non-alcoholic fatty liver disease (NAFLD) is urgently needed. In the present study, we investigated whether the Chinese medicine Chai Hu Li Zhong Tang (CHLZT) could protect against the development of NAFLD. Rats in an animal model of NAFLD were treated with CHLZT, and their serum levels of cholesterol (TC), triglycerides (TG), high density lipoprotein-cholesterol (HDL-C), low density lipoprotein-cholesterol (LDL-C), aspartate aminotransferase (AST), and alanine aminotransferase (ALT) were detected with an automatic biochemical analyzer. A cellular model of NAFLD was also established by culturing HepG2 cells in a medium that contained a long chain fat emulsion. Those cells were treated with CHLZT that contained serum from rats. After treatment, the levels of adenylate-activated protein kinase (AMPK) α (AMPKα), p-AMPKα, acetyl coenzyme A carboxylase (ACC) α (ACCα), pACCα, PPARγ, and SREBP-2 were detected. The AMPK agonist, acadesine (AICAR), was used as a positive control compound. Our results showed that CHLZT or AICAR significantly decreased the serum levels of TG, TC, LDL-C, AST, ALT, and insulin in NAFLD rats, and significantly increased their serum HDL-C levels. Treatments with CHLZT or AICAR significantly decreased the numbers of lipid droplets in NAFLD liver tissues and HepG2 cells. CHLZT and AICAR increased the levels of p-AMPKα and PPARγ in the NAFLD liver tissues and HepG2 cells, but decreased the levels of ACC-α, p-ACC-α, SREBP-2, and 3-hydroxyl-3-methylglutaryl-coenzyme A reductase (HMGR). CHLZT protects against NAFLD by activating AMPKα, and also by inhibiting ACC activity, down-regulating SREBP2 and HMGR, and up-regulating PPAR-γ. Our results suggest that CHLZT might be useful for treating NAFLD in the clinic.

## Introduction

Non-alcoholic fatty liver disease (NAFLD) is a clinical syndrome characterized by hepatocellular steatosis, cellular injury, and the infiltration of inflammatory cells into the liver tissues of individuals without a history of excessive drinking [1,2]. Epidemiological studies indicate that the prevalence of NAFLD has surpassed those of viral hepatitis and alcoholic liver disease (ALD), making it the world’s most commonly diagnosed medical and social problem [3]. In Western countries, the prevalence of NAFLD is now two- to three-fold higher than those of hepatitis B, hepatitis C, and ALD. In Japan, the prevalence of NAFLD has increased 3–20-fold, and now exceeds that of hepatitis C. The early clinical manifestation of NAFLD is mainly simple fatty liver, which is characterized by liver steatosis and an accumulation of triglycerides (TGs) in liver tissue [4,5].

Although the pathogenesis of NAFLD remains unclear, many factors may lead to its occurrence and development [6,7], including lipid metabolism disorders, insulin resistance, oxygen free radicals, iron overload, and inflammation [8,9]. An excessive uptake of nutrients by liver tissue is the main cause of hepatic steatosis. Although the liver can store some nutrient-derived energy, excessive nutrient intact accelerates the production of fatty acids and TGs that accumulate in liver tissues [6,10]. Next, oxidative stress, lipid peroxidation, and inflammatory cytokines all help to mediate liver necrosis, inflammation, and fibrosis [4].

Adenylate-activated protein kinase (AMPK) is an important factor that regulates intracellular energy metabolism [11]. It can regulate the body’s energy metabolism by sensing changes in the ratio of AMP to ATP in the cytoplasm, and then help to maintain an adequate energy supply compared with demand balance [12,13]. When AMPK signaling molecules become activated, various pathways involved in fatty acid and TG metabolism are switched off, and metabolic pathways involved in fatty acid oxidation, glucose uptake, and glycolysis become activated [14,15].

Previous studies have confirmed that AMPK signaling molecules play a key role in hepatic lipid metabolism [12]. There are two AMPK target proteins: acetyl coenzyme A carboxylase (ACC) and the 3-hydroxyl-3-methylglutaryl-coenzyme A reductase (HMGR), respectively. ACC and HMGR both play important roles in the synthesis of fatty acids and cholesterol (TC) [16]. The phosphorylation of AMPK inhibits ACC activity and reduces the levels of malonyl-CoA in liver cells, thereby inhibiting the synthesis of fatty acids and enhancing their utilization and oxidation. These effects ultimately serve to inhibit the synthesis of TC and fatty acids in the liver [17]. Many studies have confirmed that PPAR-γ and SREBPs, such as SREBP2, play an important role in maintaining lipid homeostasis in animals [18].

The traditional Chinese herbal medicine Chai Hu Li Zhong Tang (CHLZT) is composed of Xiao Chai Hu Tang and Li Zhong Tang. Studies have shown that Xiao Chai Hu Tang is effective for reducing body fat and weight [19–21]; however, its usefulness and mechanism of action in treating NAFLD remain unclear. In the present study, we established a rat model of NAFLD by feeding rats a high-fat diet. We also established a cellular model of NAFLD by culturing HepG2 cells in a high-fat medium. We then used these models to investigate whether CHLZT might be useful for treating NAFLD, and also its effects on AMPK, PPAR-γ, and SREBP2 signaling.

## Materials and methods

### Standardized preparation of CHLZT

The starting medicinal materials Bupleurum (6 g), Scutellaria (6 g), ginger Pinellia (9 g), Codonopsis (9 g), Atractylodes (12 g), Poria (15 g), turmeric (6 g), Zhigancao (6 g), ginger (9 g), and jujube (9 g) were identified by professional staff at the Institute of Medicine, and then verified for their specific strain, origin, and quality. These native medicines do not contain heavy metals. The herbs were thoroughly cleaned two times in five-fold volumes of tap water and then soaked for 30 min in a ten-fold volume of distilled water. They were then cooked for 1 h with 700 watts of heat; after which, the water was removed by filtration. The residue was then cooked a second time at the same temperature for 40 min; after which, a six-fold volume of water was added and the residue was cooked again for 40 min. The filtrates were then combined and concentrated by heating with 700 watts of heat; this producing a liquid that contained 1 g of raw materials per ml. The liquid was sterile packaged and prepared for use.

### Animal studies

Healthy SPF male SD rats were obtained from the Experimental Animal Center of Southern Medical University and assigned to four groups: a normal group, NAFLD group, CHLZT treatment group, and an AMPK agonist AICAR treatment group (*n*=10 rats per group). To establish the NAFLD rat model, rats were fed a high-fat diet (88% common feed + 2% TC + 10% lard) for 8 weeks after adaptive feeding for 1 week. Rats in the normal group were fed a normal diet. Rats in the CHLZT group received a daily intragastric CHLZT (3.5 ml/day) and were fed a high-fat diet from week 9 through week 12 of the study. Rats in the AICAR treatment group received a daily intraperitoneal injection of AICAR (25 mg/kg/day) that was initially prepared in DMSO and then further diluted with sterile saline to the desired concentration. Those rats were also fed the high-fat diet [22,23]. After 12 weeks, all the rats were killed by cervical dislocation, and their blood and liver tissues were collected. The study protocol was approved by the Institutional Animal Care and Use Committee of Shanxi University of Chinese Medicine.

### Serum levels of TC, TG, low density lipoprotein-cholesterol, high density lipoprotein-cholesterol, alanine aminotransferase, and aspartate aminotransferase

After 8 weeks of continuous treatment, the rats were fasted for 12 h, after which, blood was collected from the abdominal aorta of each rat and the serum was separated for analysis. Serum TC, TG, low density lipoprotein-cholesterol (LDL-C), high density lipoprotein-cholesterol (HDL-C), alanine aminotransferase (ALT), and aspartate aminotransferase (AST) levels in five randomly selected rats in each group (others for ELISA) were detected with an automatic biochemical analyzer.

### Cell culture and treatment

HepG2 cells (ATCC, Manassas, VA, U.S.A.) were cultured in 79% Dulbecco’s modified Eagle’s medium (DMEM, 10569044, Gibco, Waltham, MA, U.S.A.) that contained 20% FBS (10099-141, Gibco) and 1% penicillin-streptomycin (100 U/ml, 15140-122, Gibco) [24]. Other groups of HepG2 cells were cultured in medium that contained a 1% long chain fat emulsion (0338-0519-48, Intralipid® 20%, Baxter, Deerfield, IL, U.S.A.) for 48 h; those cells were used to construct the NAFLD cell model [25]. Next, the cells were treated with either CHLZT-containing serum (defined as Compound-S) or AICAR-containing serum (defined as AICAR-S) at final concentrations of 20%. Control cells were incubated with normal FBS (20%).

### Oil Red O staining

The pieces of liver tissue were separated and stored in liquid nitrogen at −80°C. Sections of tissue and aliquots of HepG2 cells were washed three times with PBS and then fixed with 10% formaldehyde for 1 h at room temperature. Next, the tissue sections and cells were incubated with Oil Red O for 30 min, rinsed three times with distilled water, and then observed under an inverted microscope. ImagePro Plus 7 software (Media Cybernetics, Inc., Rockville, MD, U.S.A.) was used to quantitate the amount of Oil Red O staining.

### Immunohistochemistry

Pieces of liver tissue were cut into 4-µm sections and their endogenous enzymes were inactivated by incubation in 3% hydrogen peroxide for 10 min. Antigens were then retrieved by immersing the sections in a citrate buffer solution. After being heated in a microwave oven for 4 min, the sections were cooled, washed with PBS for 5 min, and then blocked with 5% BSA for 20 min at room temperature. Next, the sections were incubated overnight at 4°C with 50 µl of primary antibody against p-AMPK (Abcam, Cambridge, MA, U.S.A.), followed by incubation with an HRP–conjugated secondary antibody. After nuclear staining, the sections were dehydrated in a graded series of ethanol solutions (70, 80, 90, and 100%, for 5 min each), and then mounted with neutral gum on to dry slides. Images were taken under an optical microscope. Aliquots of HepG2 cells were fixed in 4% glutaraldehyde for 15 min, and then blocked with 2% BSA for 30 min. The cells were then permeabilized for 5 min with 0.05% Triton-X100 and incubated overnight with anti-p-AMPK (Abcam, U.S.A.) at 4°C. Next, the cells were incubated for 1 h at room temperature with Alexa Fluor 488–conjugated secondary antibody (Boster, China) and then mounted using Vectashield mounting medium (Vector Labs, Burlingame, CA, U.S.A.). Images were obtained by confocal microscopy (Leica, Germany) and ImagePro Plus 7 software was used to quantitate the amount of immunostaining.

### ELISA

After treatment, the levels of HMGR and insulin were examined using ELISA kits (Elabscience, Houston, TX, U.S.A.) according to manufacturer’s instructions. After being incubated with biotinylated antibody (100 μl/well) for 1 h at 37°C, the culture plates were washed five times with PBS, incubated with the appropriate enzyme substrate at 37°C for 30 min, and then incubated in the dark with a chromogenic substrate (3,3′,5,5′-Tetramethylbenzidine (TMB)) solution for 15 min. The staining reactions were terminated with 100 μl of STOP solution. The optical density of each well at 450 nm was read with a microplate reader.

### qRT-PCR

After treatment, the total RNA in bone marrow-derived macrophages (BMMs) was isolated using TRIzol reagent (Invitrogen, Carlsbad, CA, U.S.A.) according to a standard protocol. RT-qPCR was performed using SYBR Premix Ex Taq™ (Takara, Shiga, Japan) and a Real-Time PCR System (Applied Biosystems, Santa Clara, CA, U.S.A.) according to the manufacturer’s instructions. The primers used are shown in Table 1. All tests were performed in triplicate. Gene expression was normalized to GAPDH expression, and calculated using the 2^−ΔΔ*C*^_t_ method.

**Table 1:** Sequence of the primers for qRT-PCR

Gene	Forward sequence (3′–5′)	Reverse sequence (3′–5′)
Rat-*PPARγ*	TACCACGGTTGATTTCTCCA	TGAGGGAGTTTGAAGGCTCT
Rat-*SREBP-2*	GGGACATCGACGAGATGCTA	AATGGGACCTGGCTGAATGA
Rat-*GAPDH*	CCTCGTCTCATAGACAAGATGGT	GGGTAGAGTCATACTGGAACATG
Human-*PPARγ*	ACCAAAGTGCAATCAAAGTGGA	ATGAGGGAGTTGGAAGGCTCT
Human-*SREBP-2*	CCTGGGAGACATCGACGAGAT	TGAATGACCGTTGCACTGAAG
Human-*GAPDH*	TGTTCGTCATGGGTGTGAAC	ATGGCATGGACTGTGGTCAT

### Western blot studies

The total proteins of liver tissues (four rats randomly selected from each group) and cells were extracted from BMMs using RIPA lysis buffer and then quantitated with a Pierce BCA Protein Assay Kit (Thermo Fisher, Waltham, MA, U.S.A.). The proteins were separated by 10% SDS/PAGE and then transferred on to a nitrocellulose membrane, which was then blocked with 5% skim milk. The membrane was then incubated with primary antibodies that included anti-AMPK, anti-p-AMPK, anti-ACC, anti-p-ACC, anti-PPARγ, and anti-SREPBP-2 (Abcam, U.S.A.) overnight at 4°C, and then incubated with a HRP–conjugated secondary antibody (Bioworld, China) for 1 h at room temperature. The immunostained proteins were visualized with ECL-Plus reagent (Millipore, Billerica, MA, U.S.A.).

### Statistical analysis

All experiments involving cell detection were performed in triplicate, and all serum and tissue assays were repeated three times. Data were presented as the mean ± S.D. All data analyses were performed using SPSS 16.0 software (SPSS UK, Ltd., Woking, U.K.). Student’s *t* test was used for comparisons of two groups, and one-way ANOVA with SNK was used for multiple group comparisons. A *P*-value < 0.05 was considered statistically significant.

## Results

### The effects of CHLZT on serum levels of lipids, AST, ALT, and insulin in NAFLD rats

**Figure 1:**
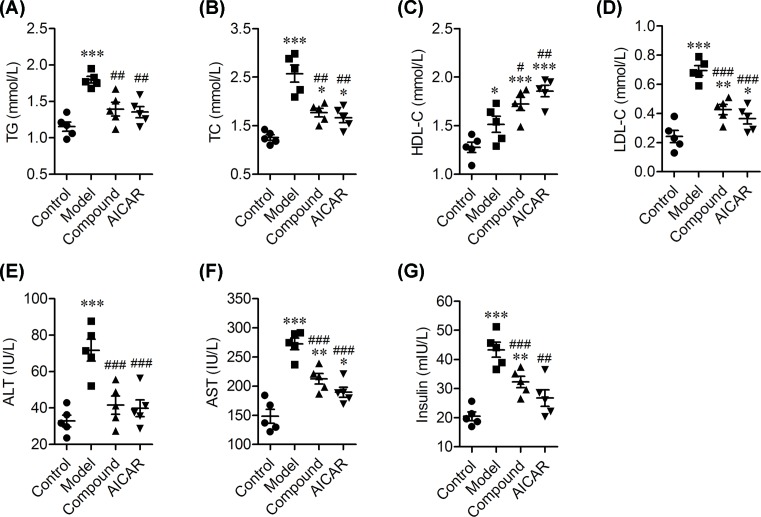
Serum levels of lipids and insulin in NAFLD rats NAFLD model rats (*n*=10) were treated for 8 days with CHLZT or AICAR and their serum levels of lipids, liver enzymes, and insulin were detected with an automatic biochemical analyzer after they had fasted for 12 h. The levels of TG (**A**) TC (**B**), HDL-C (**C**), LDL-C (**D**), ALT (**E**), AST (**F**), and insulin (**G**) in different groups (*n*=5). *P<0.05, **P<0.01, ***P<0.001 compared with control. ^#^*P*<0.05, ^##^*P*<0.01, ^###^*P*<0.001 compared with model.

To evaluate our NAFLD rat model and the effects of CHLZT, we detected the levels of TG, TC, HDL-C, LDL-C, ALT, AST, and insulin in the model rats (Figure 1). When compared with rats in the control group, the TG levels in the model group were significantly increased; however, they did not significantly differ from those in the CHLZT and AICAR groups (Figure 1A). Treatment with CHLZT or AICAR significantly decreased the levels of TG when compared with those in the model group (Figure 1A). The TC levels in the model, CHLZT, and AICAR groups were significantly higher than those in the control group (Figure 1B). Treatment with CHLZT or AICAR significantly decreased the TC levels when compared with those in the model group (Figure 1B). The HDL-C levels in the model, CHLZT, and AICAR groups were significantly increased when compared with those in the control group (Figure 1C). CHLZT or AICAR treatment significantly increased the HDL-C levels when compared with those in the model group (Figure 1B). The LDL-C levels in the model, CHLZT, and AICAR groups were significantly higher than those in the control group (Figure 1D). CHZT or AICAR treatment significantly decreased the LDL-C levels when compared with those in the model group (Figure 1D). The ALT levels in the model group were significantly higher than those in the control group (Figure 1E). CHLZT or AICAR treatment significantly decreased the ALT levels when compared with those in the model group (Figure 1E). The AST levels in the model and AICAR groups were significantly higher than those in the control group (Figure 1F). CHLZT or AICAR treatment significantly decreased the AST levels when compared with those in the model group (Figure 1F).

The insulin levels in the model and CHLZT groups were significantly higher than those in the control group (Figure 1G). Treatment with CHLZT or AICAR significantly decreased insulin levels when compared with insulin levels in the model group (Figure 1G). In summary, treatment with CHLZT or AICAR decreased the serum levels of TG, TC, LDL-C, AST, ALT, and insulin in NAFLD rats, and significantly increased their serum HDL-C levels, suggesting that CHLZT and AICAR can serve to protect NAFLD rats.

### Fat content in the livers of NAFLD rats decreased after CHLZT treatment

**Figure 2:**
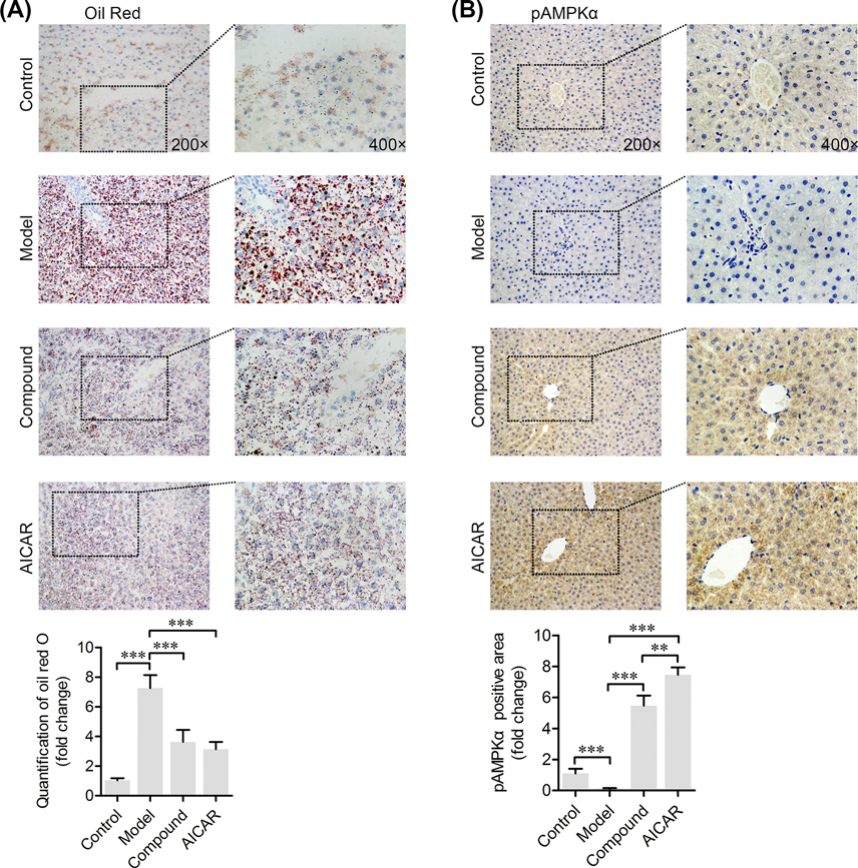
Immunohistochemistry staining of fatty substances and p-AMPKα in liver tissues The NAFLD model rats were treated with CHLZT or AICAR for 4 weeks. (**A**) The fatty substances in the liver were stained with Oil Red O. (**B**) Immunohistochemistry staining of p-AMPKα in liver tissues. **P<0.01, ***P<0.001 compared with related group.

The distribution of fat in specimens of rat liver was observed after Oil Red O staining (Figure 2A). Lipid droplets in liver tissues were stained red and distributed throughout the cell cytoplasm. The liver sections of control rats contained significantly fewer fat droplets than the liver sections of model rats. Treatment with CHLZT or AICAR significantly decreased the number of lipid droplets in the NAFLD model rats.

### CHLZT induced phosphorylation of AMPKα in NAFLD rats

Immunohistochemistry (IHC) staining for p-AMPKα was performed to explore the role of AMPKα in CHLZT-treated NAFLD rats (Figure 2B). Specimens of liver tissue that were positive for p-AMPKα displayed a brown color throughout the cytoplasm. When compared with p-AMPKα staining in the control specimens, the amount of p-AMPKα staining in specimens from the model group was significantly decreased. This indicated that the CHLZT and AICAR treatment had significantly increased the amount of p-AMPKα in the liver tissues of NAFLD model rats.

### CHLZT induced expression of p-AMPKα and PPARγ, but inhibited expression of ACC-α, p-ACC-α, SREBP-2, and HMGR in NAFLD rats

**Figure 3:**
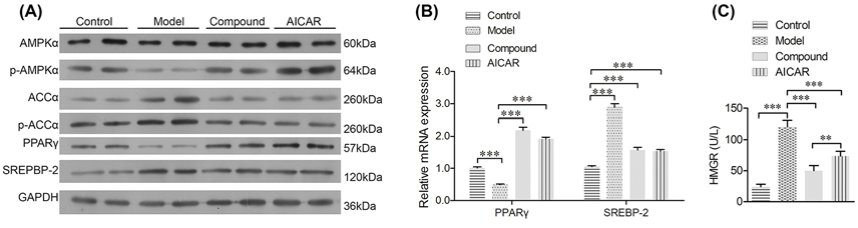
Expression of AMPKα, p-AMPKα, PPARγ, ACC-α, p-ACC-α, SREBP-2, and HMGR in liver tissues The NAFLD model rats were treated with CHLZT or AICAR for 4 weeks. (**A**) Western blot detection of AMPKα, p-AMPKα, ACC-α, p-ACC-α, PPARγ, and SREBP-2 (*n*=4). (**B**) qRT-PCR detection of PPARγ and SREBP-2 in the different groups (*n*=3). (**C**) ELISA for HMGR activity (*n*=5). **P<0.01; ***P<0.001.

To detect the downstream signaling of AMPKα, the levels of PPARγ, ACC-α, p-ACC-α, and SREBP-2 in rats were detected. Consistent with results of IHC staining for p-AMPKα, the levels of p-AMPKα in the model group were significantly lower than those in the control group. CHLZT or AICAR treatments significantly increased the levels of p-AMPKα in the NAFLD model rats (Figure 3A). Moreover, the levels of ACC-α and p-ACC-α in the model rats were significantly higher than those in the control rats. CHLZT or AICAR treatments significantly reduced the ACC-α and p-ACC-α levels in the NAFLD model rats (Figure 3A). When compared with levels in control rats, the levels of *PPARγ* mRNA and protein were significantly decreased in the model rats. CHLZT or AICAR treatments significantly reduced the levels of *PPARγ* mRNA and protein in the NAFLD model rats (Figure 3A,B). The levels of *SREBP-2* mRNA and protein in the model rats were significantly higher than those in the control rats. CHLZT or AICAR treatments significantly increased the levels of *PPARγ* mRNA and protein in NAFLD model rats (Figure 3A,B). HMGR levels in the model rats were significantly higher than those in control rats (Figure 3C). CHLZT or AICAR treatments significantly reduced the HMGR levels in NAFLD model rats.

### CHLZT inhibited the aggregation of lipids, but induced the phosphorylation of AMPKα in NAFLD cells

**Figure 4:**
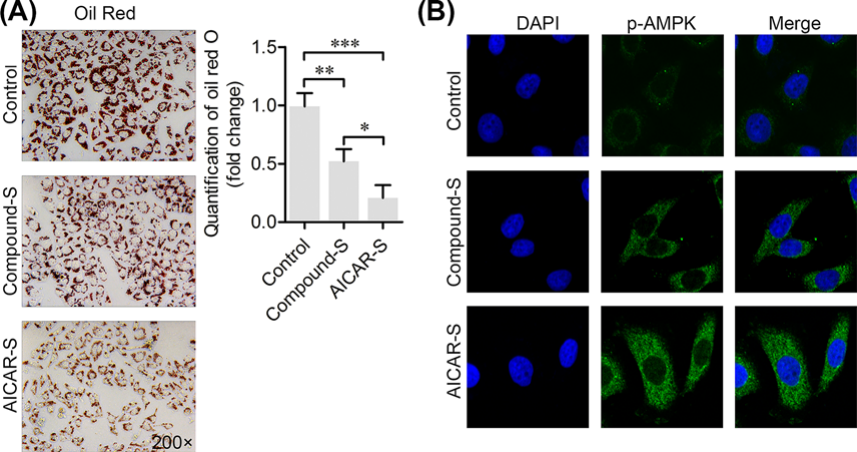
Effect of CHLZT-containing serum on lipid aggregation and phosphorylation of AMPKα in NAFLD cells The NAFLD cell model was established by treating HepG2 cells with medium containing a long chain fat emulsion. The NAFLD cells were treated with CHLZT-containing serum or AICAR. (**A**) Oil Red O staining. (**B**) Immunofluorescence staining of p-AMPKα in cells. *P<0.05, **P<0.01, ***P<0.001 compared with related group.

HepG2 liver cells were cultured in medium containing a long chain fat emulsion to establish the NAFLD cell model. After the NAFLD model cells were treated with CHLZT-containing serum or AICAR, Oil Red O staining showed that those treatments had inhibited lipid aggregation in the cell cytoplasm (Figure 4A). The phosphorylation of AMPKα in cells was detected by immunofluorescence staining (Figure 4B). Treatment with CHLZT-containing serum or AICAR induced the phosphorylation of cytoplasmic AMPKα.

### CHLZT induced expression of p-AMPKα and PPARγ, but inhibited expression of ACC-α, p-ACC-α, SREBP-2, and HMGR in NAFLD cells

**Figure 5:**
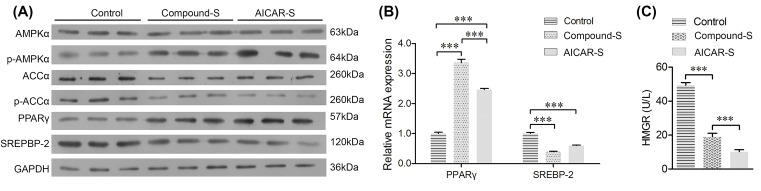
Expression of AMPKα, p-AMPKα, PPARγ, ACC-α, p-ACC-α, SREBP-2, and HMGR in NAFLD cells The NAFLD cells were treated with CHLZT or AICAR. (**A**) Western blot detection of AMPKα, p-AMPKα, ACC-α, p-ACC-α, PPARγ, and SREBP-2. (**B**) qRT-PCR detection of PPARγ and SREBP-2. (**C**) ELISA for HMGR. ***P<0.001.

The levels of PPARγ, ACC-α, p-ACC-α, and SREBP-2 in NAFLD cells were also detected (Figure 5A). When compared with their levels in control cells, the levels of p-AMPKα and PPARγ in cells treated with CHLZT-containing serum or AICAR were significantly increased. ACC-α and p-ACC-α levels were significantly reduced by treatments with CHLZT-containing serum or AICAR (Figure 5A). Moreover, the levels of *PPARγ* mRNA and protein were significantly increased by the CHLZT-containing serum and AICAR treatments, while the levels of *SREBP-2* mRNA and protein were significantly reduced by those treatments (Figure 5A,B). CHLZT or AICAR treatments also significantly reduced the HMGR levels in NAFLD model cells (Figure 5C).

## Discussion

**Figure 6:**
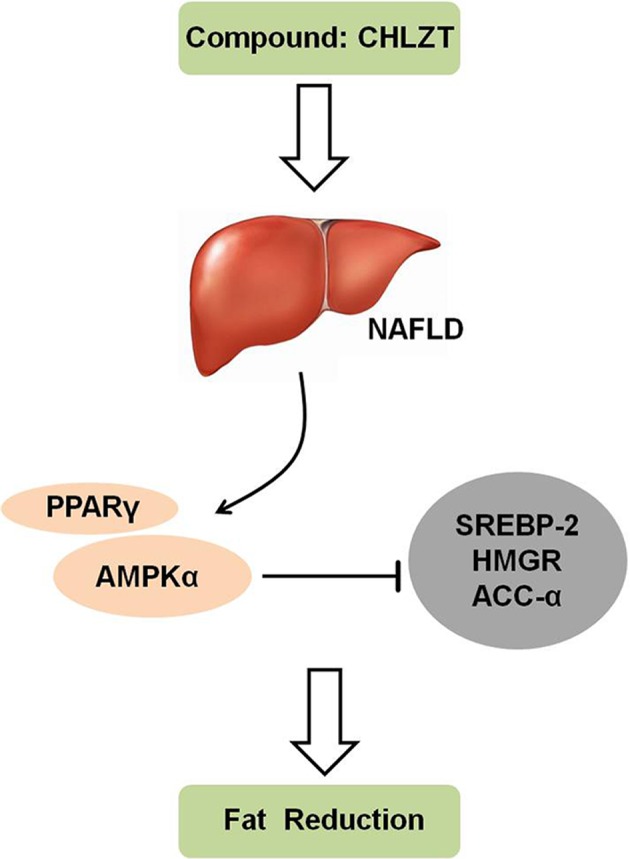
The Chinese medicine CHLZT was effective in protecting against NAFLD Schematic diagram showing how the Chinese medicine CHLZT reduces lipid levels in NAFLD by enhancing PPARγ expression, promoting the phosphorylation of AMPKα, and inhibiting SREBP-2 expression, the phosphorylation of ACC-α, and HMGR activity.

In the present study, we treated NAFLD rats with CHLZT and then analyzed their blood’s biochemical parameters to determine how the affects produced by CHLZT might be related to AMPK, PPAR-γ, SREBP2, and signaling. The disease spectrum of NAFLD varies with its stage of progression, which includes four stages of pathology: simple fatty liver, non-alcoholic steatohepatitis, fatty liver fibrosis, and fatty liver cirrhosis [26,27]. Steatohepatitis is a relatively common stage of the disease spectrum, and is characterized by an accumulation of TGs [27,28]. Steatohepatitis an important intermediate step in the transition from fatty liver to liver fibrosis or cirrhosis [29]. Moreover, the levels of TG, AST, ALT, and LDL-C are closely associated with NAFLD [26,27]. The TG levels in rats fed a high-fat diet or in cells cultured in medium containing a long chain fat emulsion were remarkably increased, and were accompanied by increased AST, ALT, LDL-C, and insulin levels in liver tissues, suggesting the successful establishment of NAFLD rat and cell models. CHLZT significantly down-regulated the TG content in NAFLD rats, and reduced the levels of AST, ALT, LDL-C, and insulin in liver tissues. Furthermore, the aggregation of lipids in both NALFD rats and cells was significantly inhibited by CHLZT. These results indicated that the Chinese medicine CHLZT was effective in protection against NAFLD (Figure 6).

ACC is a rate-limiting enzyme involved in the synthesis of fatty acids. The acetyl coenzyme A generated by glucose metabolism is used to synthesize malonyl-CoA via the action of ACC [30]. Malonyl-CoA is the primary product of fat synthesis. It can inhibit the activity of carnitine palmitate transferase-1 through a negative feedback mechanism, and thereby inhibit ketone body formation and fatty acid oxidation. The rate-limiting enzyme in TC synthesis is HMGR, which catalyzes the production of mevalonate by hydroxymethylglutaryl CoA [31]. AMPKα inhibits the activity of ACC, and thereby reduces the levels of malonyl-CoA in liver cells. This reduction in malonyl-CoA content inhibits the synthesis of fatty acids, enhances the utilization and oxidation of fatty acids, and ultimately inhibits the synthesis of TC and fatty acids in the liver [17]. Activation of AMPKα signaling not only reduces the synthesis of TC and fat, but also enhances the oxidation of fatty acids and reduces the concentrations of free fatty acids in blood, which has a certain therapeutic significance for NAFLD [32]. A variety of adipokines are involved in the pathogenesis of NAFLD [33,34]. Adiponectin can reduce the synthesis of fatty acids and enhance β-oxidation of fatty acids by activating AMPKα and increasing the sensitivity of cells to insulin [35–38]. Similar to AICAR, CHLZT induced the activation of AMPKα, but reduced ACC activity and insulin levels in both NAFLD rats and cells, suggesting that CHLZT protects against NAFLD by activating AMPKα and inhibiting ACC activity.

PPAR-γ decreases insulin resistance by regulating insulin sensitivity and promoting the synthesis of adiponectin [39,40]. As a key enzyme involved in fatty acid oxidation, PPAR-γ regulates all aspects of fatty acid metabolism and promotes the uptake of free fatty acids and the storage of TGs [41]. In addition, PPAR-γ induces adipocyte differentiation, and its expression increases with the differentiation of adipocytes [42]. We found that CHLZT increased PPAR-γ levels, which might contribute to the differentiation of adipocytes. Many studies have confirmed that SREBPs, such as SREBP2, not only play an important role in regulating fatty acid metabolism, but also help to alleviate autophagy dysfunction and participate in the synthesis and absorption of TC, phospholipids, and other substances, so as to maintain the homeostasis of lipids in animals [43]. We found that CHLZT reduced the SREBP2 levels in NAFLD liver tissues and cells.

In conclusion, CHLZT protects against NAFLD by activating AMPKα, inhibiting ACC activity, down-regulating SREBP2 and HMGR, and up-regulating PPAR-γ. Our findings may help to improve the clinical outcomes of patients with NAFLD.

## Abbreviations

ACC: acetyl coenzyme A carboxylase
ALD: alcoholic liver disease
ALT: alanine aminotransferase
AMPK: adenylate-activated protein kinase
AST: aspartate aminotransferase
ATCC: American Type Culture Collection
BMM: bone marrow-derived macrophage
CHLZT: Chai Hu Li Zhong Tang
GAPDH: glyceraldehyde-3-phosphate dehydrogenase
HDL-C: high density lipoprotein-cholesterol
HMGR: 3-hydroxyl-3-methylglutaryl-coenzyme A reductase
HRP: horseradish peroxidase
IHC: immunohistochemistry
LDL-C: low density lipoprotein-cholesterol
NAFLD: non-alcoholic fatty liver disease
PPAR: peroxisome proliferator activated receptors
RIPA: Radio-immunoprecipitation Assay
qRT-PCR: quantitative reverse transcription -polymerase chain reaction
SD: standard deviation
SNK: Student-Newman-Keuls
SREBP-2: sterol regulatory element binding protein 2
SRF: serum response factor
TC: cholesterol
TG: triglyceride
